# Assessment of Coastal Ecosystem Services for Conservation Strategies in South Korea

**DOI:** 10.1371/journal.pone.0133856

**Published:** 2015-07-29

**Authors:** Min Gon Chung, Hojeong Kang, Sung-Uk Choi

**Affiliations:** School of Civil and Environmental Engineering, Yonsei University, Seoul, Korea; Shandong University, CHINA

## Abstract

Despite the fact that scientific and political consideration for ecosystem services has dramatically increased over the past decade, few studies have focused on marine and coastal ecosystem services for conservation strategies. We used an ecosystem services approach to assess spatial distributions of habitat risks and four ecosystem services (coastal protection, carbon storage, recreation, and aesthetic quality), and explored the tradeoffs among them in coastal areas of South Korea. Additionally, we analyzed how the social and ecological characteristics in coastal areas interact with conservation and development policies by using this approach. We found strong negative associations between the habitat risks and ecosystem services (aquaculture, carbon storage, recreation, and aesthetic quality) across the coastal counties. Our results showed that the intensity of the habitat risks and the provision of ecosystem services were significantly different between reclamation-dominated and conservation-dominated counties, except for coastal vulnerability. A generalized linear model suggested that reclamation projects were dependent on economic efficiency, whereas demographic pressures and habitat conditions influenced the designation of protected areas at a county level. The ecosystem services approach provided guidelines to achieve both sustainable development and environment conservation. By using the approach, we can select the priority areas for developments while we can minimize the degradation of biodiversity and ecosystem services. As cultural ecosystem services are evenly distributed throughout coastal areas of South Korea, decision makers may employ them to improve the conditions of coastal wetlands outside of protected areas.

## Introduction

Ecosystem services are defined as benefits to human well-being that are derived from nature systems [1, 2]. The concept of ecosystem services deals with both scientific activities and environmental policies such as the Convention on Biological Diversity (CBD) and the Intergovernmental Science-Policy Platform on Biodiversity and Ecosystem Services (IPBES) [3]. Marine and coastal ecosystems provide a broad range of ecosystem goods and services to human society, because they are one of the most productive ecosystems on Earth [2]. Recently, most services provided by the oceans and coastal zones have more rapidly deteriorated than other ecosystems [4]. Population distribution analysis indicates that about two billion people live in 7.6% of the land areas, and the coasts of Asia have experienced particularly high population pressures [4]. This leads not only to high population densities but also to development pressures in the coastal zone.

As many studies have provided hidden values of coastal wetlands such as habitats for migratory birds, storm protection, erosion control, water treatment, and high biodiversity [5], the estimated values of coastal wetlands have increased from 14,000 $/ha/yr in 1997 to 194,000 $/ha/yr in 2014 [6]. This highlights the fact that coastal wetlands are one of the most valuable ecosystems in the world. However, coastal wetlands have been severely affected by anthropogenic impacts, which include not only *in situ* development such as land reclamation, but also pollutions derived from upland agriculture and industry [2, 4]. Therefore, it is necessary to consider terrestrial and coastal areas as a single spatial unit to assess accurately ecosystem services and implement efficient conservation strategies for coastal wetlands.

In East Asia, the reclamation projects of coastal wetlands in the past few decades have often been performed to expand land areas for increasing human activities such as urbanization and industrial development [7, 8]. Whereas many decision makers believe that reclamation is a cost-effective way to pursue economic growth by building industrial complexes on reclaimed areas, large-scale reclamation has caused the degradation of coastal ecosystems and their services [5]. Reclamation of coastal wetland has led to permanent changes in ecosystem processes and functions [9] and it reduces not only ecosystem services but also human well-being in coastal areas [10]. Since coastal wetlands are directly affected by anthropogenic land-based activities and are closely linked to human society (e.g., fisheries, recreation, and reclamation) [9], societal characteristics (i.e., demographic and socioeconomic factors) impact conservation and development strategies. Conventionally, East Asian countries performed environmental impact assessments and cost-benefit analyses for coastal wetland reclamation projects. Although the assessments and analyses could consider the reclamation impacts on fisheries and direct environmental pollution, they have not quantified biodiversity loss and its impacts on ecosystem services provided by coastal wetlands [5, 9].

Implementing reclamation policies often causes conflicts between different groups, which demand either conservation or development [11]. If development pressure is extremely high, politicians might be averse to implement conservation policies due to the conservation/development tension [12]. The designation of protected areas is a primary tool for relieving the pressures of coastal zone development among conservation strategies [13]. However, stakeholders often oppose the designation of protected areas, because it will restrict the use of provisioning services like fishery activities required for sustainable livelihoods. Although decision makers have recently attempted to plan compensation arrangements for protected areas to avoid conflicts between stakeholders and governments [14], previous studies could not provide scientific evidence for decision-making processes to resolve the conflicts. Only a few studies have considered the synergy of biodiversity and ecosystem services along with societal characteristics within coupled human and natural systems (CHANS) [15], particularly in coastal areas.

An ecosystem services approach provides a way to integrate ecosystem services and socioeconomic characteristics into decision-making processes [16]. The state-of-art of ecosystem services approach largely consists of scientific assessment tools such as spatial modeling, tradeoff analysis, and economic valuation and environmental policy instruments such as regulation and payment for ecosystem services. Therefore, using the ecosystem services approach in coastal areas is essential to not only assess biodiversity and ecosystem services but also involve socioeconomic characteristics for conservation strategies. The ecosystem services approach can help us prioritize coastal areas for conservation and assist decision makers in informing the configuration of a spatial plan in coastal areas.

In this paper, we aimed to reveal determinants behind wetland reclamation projects and the designation of protected areas in coastal zones of South Korea. Additionally, we aimed to propose effective strategies for coastal management using an ecosystem services approach. We followed a scientific assessments tool, InVEST model, to understand the complex interactions between human and natural systems. Then, we performed spatial assessments of natural habitat risks and ecosystem services. Using the results of the spatial assessments, we carried out tradeoff analysis and uncovered the determinants of reclamation projects and the designation of protected areas.

## Materials and Methods

### Study areas

**Fig 1:**
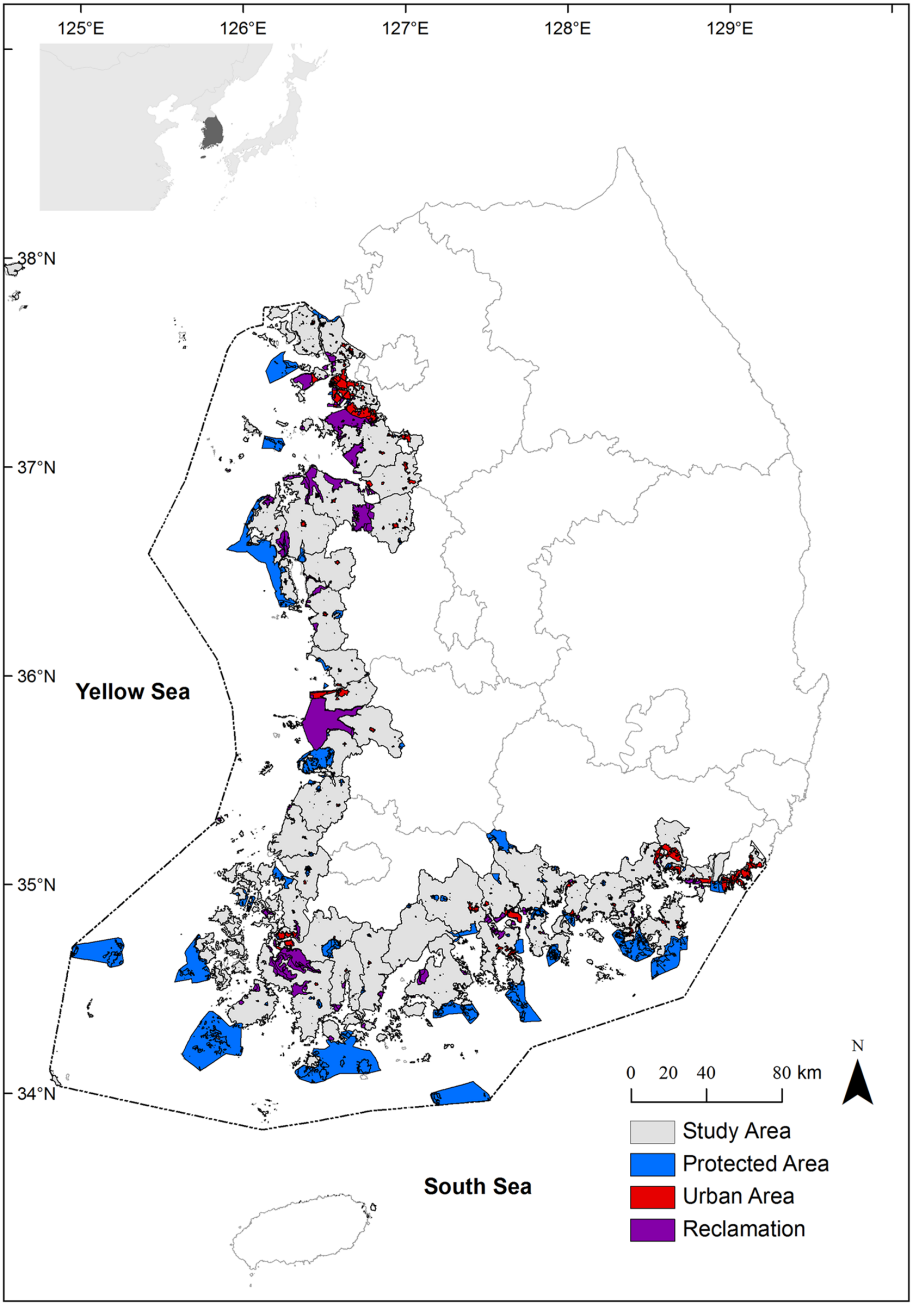
Distribution of study areas, protected areas, urban areas and reclaimed areas [Data Source: [19–22]]

Study areas consist of the western and southern coastal zones of South Korea, which cover both terrestrial counties and coastal areas (Fig 1). The coastal areas comprise 61 counties with sizes ranging from 3 to 1,006 km^2^ in area. It also contains 27% of the total population of Korea. The average annual temperatures are between 10 and 15°C, and the annual precipitation varies from 1,200 to 1,500 mm in the central region and from 1,000 to 1,800 mm in the southern region [17]. The sea level change between high and low tide is 256 cm (ranging from 73 to 462 cm)[18].

Coastal wetlands in South Korea provide multiple ecosystem services such as fish production, coastal protection, and recreation to human society, which contribute to local livelihoods and economies [23, 24]. About 90% of total fishery licenses are issued in coastal areas, and 92% of fishery households are located in coastal counties [25]. The Korean government has conducted large-scale reclamation of coastal ecosystems for agricultural lands, urbanization, and industrial development since 1970s [8]. The urban areas of coastal counties have rapidly increased twice from 561 km^2^ in the 1980s to 1,222 km^2^ in the 2000s [21]; large-scale reclamation projects have reclaimed 754 km^2^ of coastal areas since 1982 [26]. The coastal protected areas cover 10,007 km^2^ of South Korea, which is 10% of the land area and 15% of territorial waters of South Korea [27] (Fig 1). Specifically, since over a half of coastal wetlands in South Korea have been reclaimed and developed for urbanization and industrial complexes during the 20th century [2], many remained coastal wetlands locates near urbanized and industrialized areas. In the present, the conservation areas of coastal wetlands cover about 10% or 213 km^2^ of the total coastal wetlands (2,487 km^2^) [27].

The Korean government amended the Coast Management Act in 2009 to achieve effective conservation and sustainable development of coastal areas. In 2011, the Ministry of Land, Transport and Maritime Affairs (the Ministry of Oceans and Fisheries as reconstituted in 2013) established the second integrated coastal management plan for 2011–2021 under the provisions of Article 8 of the Coast Management Act [26]. The objectives of the management plan aim not only to conserve coastal biodiversity but also to provide provisioning and cultural ecosystem services to the human society through ecosystem-based management. The Coast Management Act states that local governments will establish local integrated coastal management at a county level following the guidelines of the second integrated management plan, and therefore local governments have individualized coastal management authority.

### Selection of ecosystem services

In South Korea, anthropogenic impacts such as urbanization and industrial complexes have caused the degradation of natural habitats and biodiversity loss in coastal zones. Coastal wetland reclamation projects have dramatically degraded natural habitat and therefore led to biodiversity loss. The biodiversity loss has a negative impact on several ecosystem services [28]. Thus, we firstly assessed and mapped coastal and terrestrial habitat risks in South Korea as the proxies of biodiversity.

Conservation strategies, especially for strictly protected areas, benefit regulation and cultural services [29], whereas these strategies have restricted the use of provisioning services [30]. The second integrated management plan in South Korea was also established to conserve not only biodiversity but also regulation and cultural services, particularly in coastal areas. Although the decision makers identified the benefits of regulation and cultural services, few ecosystem service assessments support these strategies. In particular, ecosystem services research has overlooked the assessments of regulation services because these services are not utilized directly and hard to be quantified [1]. However, they may be the main indicators of regime shift risk, and therefore, it is necessary to assess regulating services at multiple scales [31].

The focus of the spatial assessment was four regulation and cultural ecosystem services (coastal protection, carbon storage, aesthetic values, and recreation) among Marine and Coastal Ecosystem Services (MCES) (Table 1). First, the spatial assessment of risky coastal areas from heavy rainfalls and storms is necessary to identify priority areas for habitat conservation or restoration. Coastal counties in South Korea are increasingly exposed to storm-induced flooding and inundation during every summer season [17], and they may be more vulnerable due to destroying natural habitats that reduce the impact of natural disasters. Second, the spatial distribution of carbon storage helps to implement conservation policy as protected areas where the level of carbon storage is high. The capacity of carbon storage in terrestrial ecosystems largely influence global climate change [32], because the total quantities of carbon storage in terrestrial ecosystems are much larger than the quantities of carbon dioxide in the atmosphere [33]. Finally, understanding the distributions of recreational opportunities and scenic amenities in coastal areas is essential for establishing comprehensive coastal management policy. Recreational activities and local tourist attractions partially influence the regional economy of coastal counties [32], and thus the cultural ecosystem services can increase income in local communities [34].

**Table 1:** The classification of marine and coastal ecosystem services [2, 35, 36].

Provisioning	Regulating and maintenance		Cultural
Food provision	Water purification	Ocean nourishment	Symbolic and aesthetic values
Water storage and provision	Air quality regulation	Life cycle maintenance	Recreation and tourism
Biotic materials and biofuels	Coastal protection	Biological regulation	Cognitive effects
	Climate regulation		
	Weather regulation		

Additionally, we added the amounts of aquaculture production (in metric tons) as a variable for statistical analyses, because the aquaculture production represents provisioning services in coastal areas, and conservation strategies can directly and indirectly impact provisioning services. Presently, the fishery resources in South Korea have been quite well regulated under several conservation acts. Traditionally, fisheries in South Korea are government-owned, and the local communities have formed cooperatives of fishing villages and gained access by obtaining fishery licenses from the government. The Fisheries Act and Fishing Villages and Fishery Harbor Act have been enacted for increasing the fishery productions and improving the quality of life for fishing workers. The Fishing Ground Management Act and Fish Resources Management Act also designate restricted fishing areas, the period of the non-fishing season, and length limits to avoid overfishing and achieve sustainable fisheries.

### Rationale for the Ecosystem Services Approach

Previous studies have applied various approaches to assess biodiversity and ecosystem services from statistical analysis to spatial model. According to MA (2005), approaches for ecosystem service studies have largely pursued to assess ‘ecosystem condition and trend’, ‘the value of ecosystem services for human well-being’, and ‘tradeoffs in ecosystem services’ [2]. Among the evaluation methods for ecosystem service assessments, case studies that relied on surveys and interviews figure out detailed spatial and temporal contexts in particular sites. However, case studies should spend enormous time and resources with high costs, while it cannot give general solutions for regional and global challenges such as global climate change [37]. For example, drivers for ecotourism participation in Wolong Nature Reserve of China were positively associated with the high income, education, and low cropland of households [38]. As the drivers for tourism participation is highly dependent on local context, the result is hard to be adopted to different areas.

Specifically, spatial assessment and advanced statistical analysis facilitate to figure out a hidden relation between human and natural systems, because socioeconomics interact with natural systems and thus impact on the status of biodiversity and ecosystem services [39, 40]. Recent studies have sought to combine socioeconomic data with spatial data within coupled human and natural systems [41, 42], but many studies separated into spatial assessment and advanced statistical analysis at multiple scales. Mapping ecosystem services performed at global [43, 44], regional [45], and local levels [46]. In addition, researchers used advanced statistical analyzes to integrate socioeconomic data with biodiversity and ecosystem services [40, 47].

In ecosystem services approach, combining spatial assessment and statistical analysis gives new perspectives in the study areas and helps to develop the regional model of both human and nature dimensions [37]. The result of spatial assessments gives spatial explicit information about biodiversity and ecosystem services and quantified values to evaluate tradeoffs among ecosystem services. The statistical analysis estimates socioeconomic and environmental drivers that may impact on the status of biodiversity and ecosystem services. Thus, the combined approach can answer important questions for ecosystem service assessments that were suggested by Seppelt et al. (2011) [48]; biophysical realism, tradeoffs, off-site effects, and stakeholder work. The approach has been mainly adopted to forest ecosystems with remote sensing and GIS [41, 42], but not for coastal ecosystems because of data availability and greater research interests in terrestrial ecosystems. In this study, we used the combined approach in coastal ecosystems and dealt with reclamation and protection activities as deforestation and reforestation, respectively.

### Data collection

The spatial dataset consisted of geographic, ecological, and social data provided by Korean government agencies and institutes [18–22, 49]. Geographic data included the Digital Elevation Model (DEM), the county boundaries, and coastline data, and it was used as basic information for ecosystem services assessment. Ecological data consisted of coastal habitats information, seafloor characteristics, and the location of protected and reclaimed areas. Social data involve the locations of industrial complexes, populations per counties, beaches, and recreation fishing points. When raw data was paper or physical data, we converted it to raster or vector formats using digitization and georeferencing. The coordinate system used in this study was WGS 1984 UTM Zone 52N, and the vector scale was standardized to 1:20,000 and the raster data had a 30-meter resolution.

The database for statistical analyses consisted of demographic, socioeconomic, and biophysical data [18, 20, 21, 49]. We also quantified habitat risk assessments and four ecosystem services at the county level (S1–S4 Tables). Demographic data involved the population size, the population density, the number of households, the average family size, and the average age of the population. Socioeconomic data represented the characteristics of each county, such as the appraised value of land, the number of annual tourists, and Gross Regional Domestic Product (GRDP) [50].

## Methods

We calculated total protected areas in South Korea: national parks, cultural properties areas, ecological and scenic conservation areas, wildlife protection areas, and coastal wetlands conservation areas. The reclaimed areas of each county were estimated by the spatial database [19].

We selected InVEST model for the spatial assessments of coastal habitat risks and ecosystem services for two reasons. First, this model is used broadly by lots of research groups in the world such as Indonesia, Colombia, Canada, and China [16, 28]. The InVEST model was developed under Natural Capital Project to map and value various ecosystem services in both terrestrial and coastal areas. Second, the InVEST model provides various spatial sub-models particularly for coastal ecosystem services [28]. Thus, by providing spatially explicit and quantified outputs, the model specializes for coastal management plan as a scientific assessment tool [28].

Our ecosystem service approach consisted of four parts. First, we mapped the impact of human activities in both terrestrial and coastal areas and identified where human impacts are high. These proxies show the status of environmental degradation and can be used for terrestrial and coastal biodiversity, because habitat degradation significantly increase the possibility of species extinctions and then lead to biodiversity loss [51]. Second, we assessed 4 ecosystem services (two regulating services and two cultural services) by using the InVEST model, which had not been considered during the decision-making process of coastal conservation in South Korea. Then we analyzed potential tradeoffs among ecosystem services and between biodiversity and ecosystem services. Finally, we examined how demographic, socioeconomic, and biophysical factors influence the designation of protected and reclaimed areas at the county level.

### Spatial Assessments

Spatial assessments used InVEST (Integrated Valuation of Environmental Services and Tradeoffs) version 2.5.6 to assess and quantify habitat risk and ecosystem services in coastal areas [51]. We selected five sub-models: coastal habitat risk assessment, coastal vulnerability model, overlap analysis model (recreation and aesthetic quality), terrestrial habitat quality model, and carbon storage model in terrestrial areas.

#### Habitat Risk Assessment

The Habitat Risk Assessment (HRA) model produces maps of habitat risk and informs how each habitat exposes each threat or stressor in marine and coastal areas [51]. The risk of stressors that came from human activities is modeled in four steps. First, the model determines the likelihood of the exposure and consequence. ‘Exposure (E)’ exposes the habitat to the stressor, and ‘consequence (C)’ is the result of this exposure. The exposure and consequence assigns a rank from 0 to 3 to create criteria. The score of the exposure and consequence are calculated similarly to weighted average,

$$E=\frac{\sum_{i=1}^{N}\frac{e_{i}}{d_{i}w_{i}}}{\sum_{i=1}^{N}\frac{1}{d_{i}w_{i}}}$$ (1)

$$C=\frac{\sum_{i=1}^{N}\frac{c_{i}}{d_{i}w_{i}}}{\sum_{i=1}^{N}\frac{1}{d_{i}w_{i}}}$$ (2)

where *e*_*i*_ represents the exposure value, *c*_*i*_ represents consequence value, *d*_*i*_ is the data quality rank, and *w*_*i*_ is the importance weights for each criterion *i*. N is the number of criteria for each habitat [51]. Second, the model combines both the exposure and consequence values and produces a risk value (R) for each stressor-habitat combination. The risk value is that stressor *j* causes risk to habitat *i*, and R_*ij*_ is quantified using Euclidean Risk calculation [51].

$$R_{i}{}_{j}=\sqrt{{\left(E-1\right)}^{2}+{\left(C-1\right)}^{2}}$$ (3)

The third step calculates the sum of risk values for each habitat, and finally the model assesses and maps the habitats’ risk hotspots.

The habitat quality model in terrestrial areas is similar to the HRA model, except that the habitat quality model employs land use and land cover maps instead of habitat layers. The model produces a habitat quality map by integrating LULC information with threats to biodiversity. The quantification of threats on each habitat in a pixel consists of four factors: 1) the relative impact of each threat *r* (*w*_*r*_); 2) the distance between habitat and the threat in grid cell *x and y* (*i*_*rxy*_ *)*; 3) the level of legal and physical protection from disturbance ($\beta_{x}$); and 4) the relative sensitivity of each habitat to each threat (*S*_*jx*_) [51]. Then, the model calculates D_*xj*_, the total threat level in each grid cell *x*, with habitat type *j*s.

$$D_{xj}=\sum_{r=1}^{R}\sum_{y=1}^{Y_{r}}\left(\frac{w_{r}}{\sum_{r=1}^{R}w_{r}}\right)r_{y}i_{rxy}\beta_{x}S_{jr}$$ (4)

The habitat quality model produces a habitat degradation map of the current landscape. To facilitate understanding where coastal areas are vulnerable to human activities, we simply overlapped coastal and terrestrial habitat risk maps into a single map.

#### Coastal vulnerability model

The coastal vulnerability model shows the risk of coastal erosion and inundation, which can also threaten human society [51]. Whereas habitat risk assessment (HRA) model assess the risk of coastal habitats by anthropogenic activities, coastal vulnerability model estimate how anthropogenic activities can affect people’s exposure to the flooding or inundation of heavy rainfall and storms. The model creates an Exposure Index (EI) map using the ranks of seven biophysical variables: geomorphology, relief, natural habitats, net sea level change, wind exposure, wave exposure, and surge potential depth contour. Indeed, the model can be used as the proxies of coastal protection of regulating ecosystem services [51]. The exposure index calculates and represents coastal vulnerability for each coastline segment or pixel as follows [52]:

$$EI={\left(R_{Geomorpholo\text{g}y}R_{\text{Re}lief}R_{Habitats}R_{SLR}R_{WindExposure}R_{WaveExposure}R_{Surge}\right)}^{\frac{1}{7}}$$ (5)

The variables in Eq 5 are as follows: *R*_*Geomorphology*_ is the rank of geomorphology for each coastline segment, *R*_*Relief*_ is the relief ranking of each coastline segment, *R*_*Habitats*_ is a natural habitat ranking for each coastline segment, *R*_*SLR*_ is the level of net sea level change within the study site, *R*_*WindExposure*_ is the wind exposure ranking of each coastline segment, *R*_*WaveExposure*_ is the average depth of adjacent seas, and *R*_*Surge*_ is the distance ranking between the coastline and the edge of the continental margin.

#### Carbon storage model

The carbon storage model estimates the carbon stocks of four carbon pools: aboveground biomass, belowground biomass, soil organic matter and dead organic matter. Carbon storage data in terrestrial areas collected from national reports in South Korea and IPCC report [53–55] (S3 Table). The amounts of each carbon pool can be collected from preceding research based on land use and land cover types [51]. The model calculated the net amount of carbon storage in terrestrial areas. In this study, we only considered carbon storage without sequestration due to the high degree of uncertainty in estimating sequestration [56].

#### Overlap Analysis Model

The overlap analysis model allows us to identify the important areas of human use (e.g., recreation and fishing) in marine and coastal areas [28]. The model can assess and map recreation and aesthetic quality in coastal areas. In this study, we determined the spatially explicit information on six common recreational activities: swimming at the beach, sea fishing, fishing village tourism, coastal visitors’ center, yachting, and scenic viewing. In addition, the model presented the areas of high aesthetic quality with scenic viewpoints. The model calculates how many activities exist in each pixel (*i*) using an Important Score (IS):

$$IS_{i}=\frac{1}{n}\sum_{i,j}U_{ij}I_{j}$$ (6)

The variables in Eq 6 are as follows: *n* is the number of human activities using the analysis, *$U_{ij}$* is the usage of activity *j* in grid cell *i*, and $\;I_{j}$ is the inter-activity weight of activity *j* [51].

### Verification of the Spatial Assessments

To verify the accuracy of spatial assessments, we compared the spatial results with existing datasets in coastal areas provided by Korean governments and institutes. They provided the ecological grading system of coastal wetlands (7 grades) [57], the estimates of typhoon damages (5 grades) [57], the status of coastal recreation and tourism (3 grades) [19], and ecological zoning map in terrestrial areas (3 grades) [21]. In all grading systems, lower grade means a better status. Since the existing datasets were either continuous data or ranking data, we performed both Pearson correlation analysis (R) for continuous data and Spearman correlation analysis (ρ) for ranking data in R statistical software [58]. If the results of the spatial assessments positively correlate with the existing datasets, we can say that the spatial assessments achieve an accuracy and reliability.

First, the ecological grading system of coastal wetlands involved habitat diversity, species diversity, and ecological health of coastal wetlands. From 2008 to 2013, researchers evaluated the ecological conditions over the 879 points of coastal wetlands in South Korea using field works and laboratory experiments [57]. We randomly selected 360 points from the ecological grading systems and compared them with the result of the coastal habitat risk assessment to test the accuracy and reliability of the approaches we employed. Second, as the grades of typhoon damages and the grades for coastal recreation and tourism only provided at a county level, we aggregated the result of coastal vulnerability, recreation, and aesthetic quality assessments to each county. As the basic spatial unit for all statistical analyzes in this study (‘Statistical Analysis’ in methods) is a coastal county, the verification process at a county level is reasonable. Then, coastal vulnerability result was compared to the grades of typhoon damages. Also, we compared the results of recreation and aesthetic quality with the grades of coastal recreation and tourism. Finally, the ecological zoning maps represented the quality of terrestrial ecosystems [21]. We summed the areas (km^2^) of the first grade (high ecological quality) and protected areas and aggregated the results of the results of terrestrial habitat quality and carbon storage at each county. Then, we compared the total areas with the results of the habitat quality and carbon storage.

### Statistical Analysis

We performed correlation analysis between habitat risk assessments and ecosystem services and among services. The analysis was performed on each pair of two habitat risk assessments and four ecosystem services. We determined the relations of their associations and identified potential tradeoffs between habitat risk and ecosystem services using R statistical software [58].

Firstly, using zonal statistics tools in ArcGIS 10.1, coastal and terrestrial habitat assessments and four ecosystem services were quantified from the spatial assessments at each county [59]. In this quantification, coastal and terrestrial habitat risk assessments, coastal vulnerability, and terrestrial carbon storage were represented as average numeric values for each county. Recreation was represented as the number of recreational sites, and aesthetic quality was calculated as the number of scenic points per county. We quantified the number of recreation spots and aesthetic quality for each county. The areas of two cultural services cannot represent the intensity at a county level, because the areas of each county vary from 3 km^2^ to 1006 km^2^.

Then, we divided coastal counties into two groups: conservation-dominated and reclamation dominated-counties. As most counties in South Korea have focused on one side of the debate, either conservation or development due to county circumstances, we divided the counties into two groups. Potential differences in the level of habitat risks and the provision of four services were assessed between the two groups using a one-way ANOVA. Also, we used a one-way ANOVA to determine if several factors—including demographic, socioeconomic, and biophysical determinants—are different between the two groups. The ANOVA assumptions (homoscedasticity and normality) were verified, and log transformations were carried out as needed.

We fitted two generalized linear models. First, we used the Poisson distribution for GLM with log-link function, but over-dispersion occurred in the Poisson regression model. To address over-dispersion, we selected the Negative binomial distribution that is used to model count response data without the assumption that the variance equals the mean. Negative Binomial regression models wrote in the following Eqs 7 and 8,

$$\text{ln}(Y_{1})=\beta_{0}+\beta_{1}X_{1}+\beta_{2}X_{2}+\beta_{3}X_{3}+\beta_{4}X_{4}+\beta_{5}X_{5}+\beta_{6}X_{6}+\beta_{7}X_{7}+\beta_{8}X_{8}+\beta_{9}X_{9}+\beta_{10}X_{10}$$ (7)

$$\text{ln}(Y_{2})=\beta_{0}+\beta_{1}X_{1}+\beta_{2}X_{2}+\beta_{3}X_{3}+\beta_{4}X_{4}+\beta_{5}X_{5}+\beta_{6}X_{6}+\beta_{7}X_{7}+\beta_{8}X_{8}+\beta_{9}X_{9}+\beta_{10}X_{10}$$ (8)

Where *β*_i_s are the parameters to be estimated by Maximum Likelihood (ML) method. The variables in Eq 7 are as follows. Y_1_ is protected areas, Y_2_ is reclaimed areas, X_1_ is population of coastal counties, X_2_ is population density, X_3_ is the number of households, X_4_ is the average number of household members, X_5_ is the average age of population, X_6_ is the appraisal value of land, X_7_ is Gross Regional Domestic Product, X_8_ is the number of fishery households, X_9_ is the number of tourists, and X_10_ is the median slope within a kilometer radius of coastline (S5 Table).

To select the best model from the initial model, we removed non-significant predictors by using Akaike Information Criterion (AIC) and the ration of the Deviance (or Pearson Chi-square) to DF. If the sub model had the lowest AIC and the ration of the Deviance to DF was close to 1, we stopped. Because AIC is a measure of goodness of model fit, a smaller value of AIC represents a better model fit. In addition, the ratio of the Deviance to DF should be close to 1, when the model is a good fit for the data. To avoid multicollinearity problems, we checked whether the largest Condition Index (CI) in all models is greater than 10. As a result, there was no evidence of multicollinearity in the final models. All statistical analyses were performed using R and SAS 9.2 [58, 60].

## Results

### Spatial Assessments

**Fig 2:**
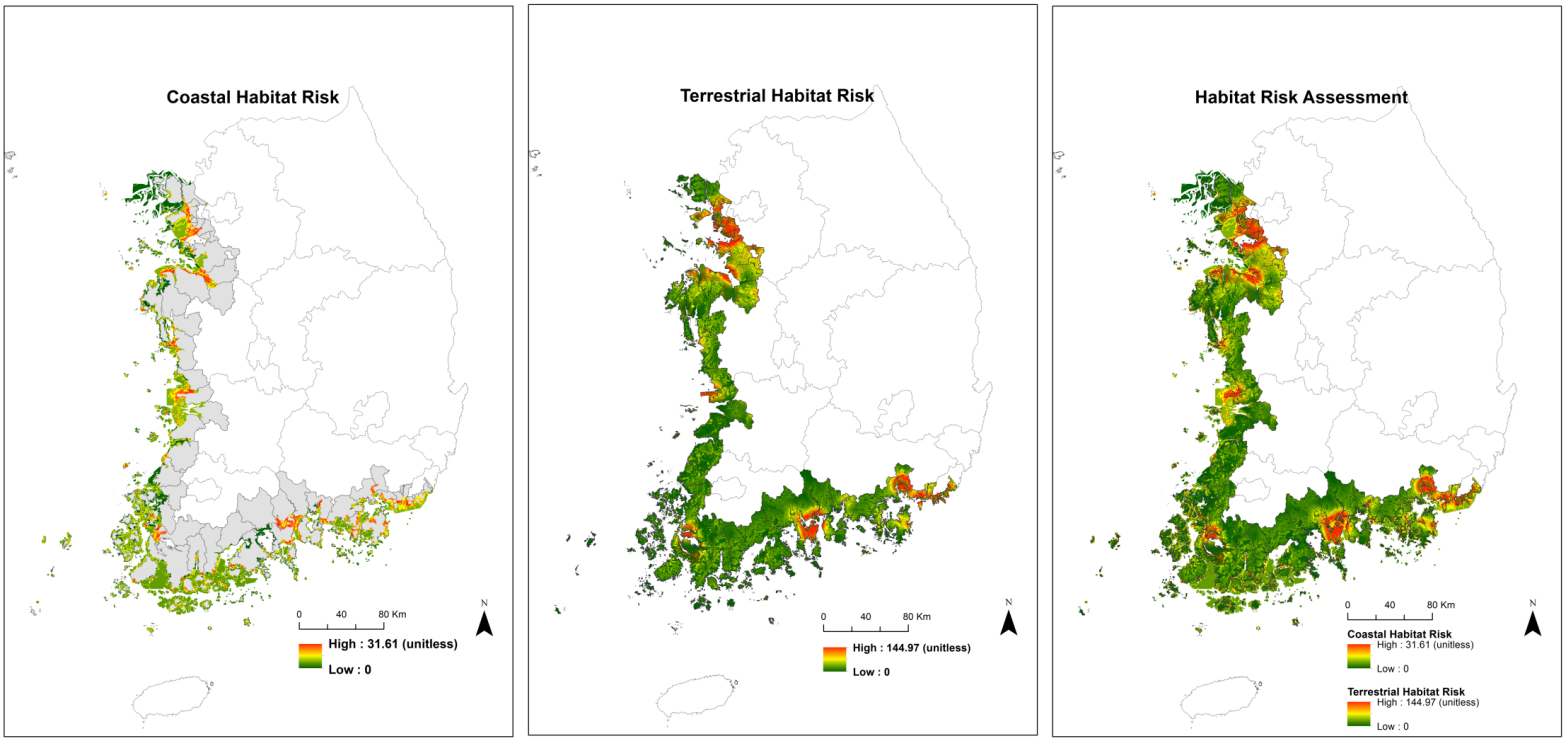
Maps of habitat risk assessments in terrestrial and coastal areas in South Korea. **Feature values on maps refer as continuous values from poor (red) to good (green) conditions**.

The coastal and terrestrial habitat risk assessments in the study area are shown in Fig 2. The distribution of the values of habitat risks was different among the counties. The habitat conditions seriously deteriorate in urbanized areas such as Incheon and Busan, but most counties in the southwest are under low levels of habitat risk. The values of coastal habitat risk strongly correlate with terrestrial habitat conditions. For example, reclaimed areas particularly lead to the degradation of natural habitats in adjacent terrestrial and coastal counties (Figs 1 and 2). The maps represent the intensities of several threats to natural habitats, while the habitat risk assessments signify the status of environmental degradation.

**Fig 3:**
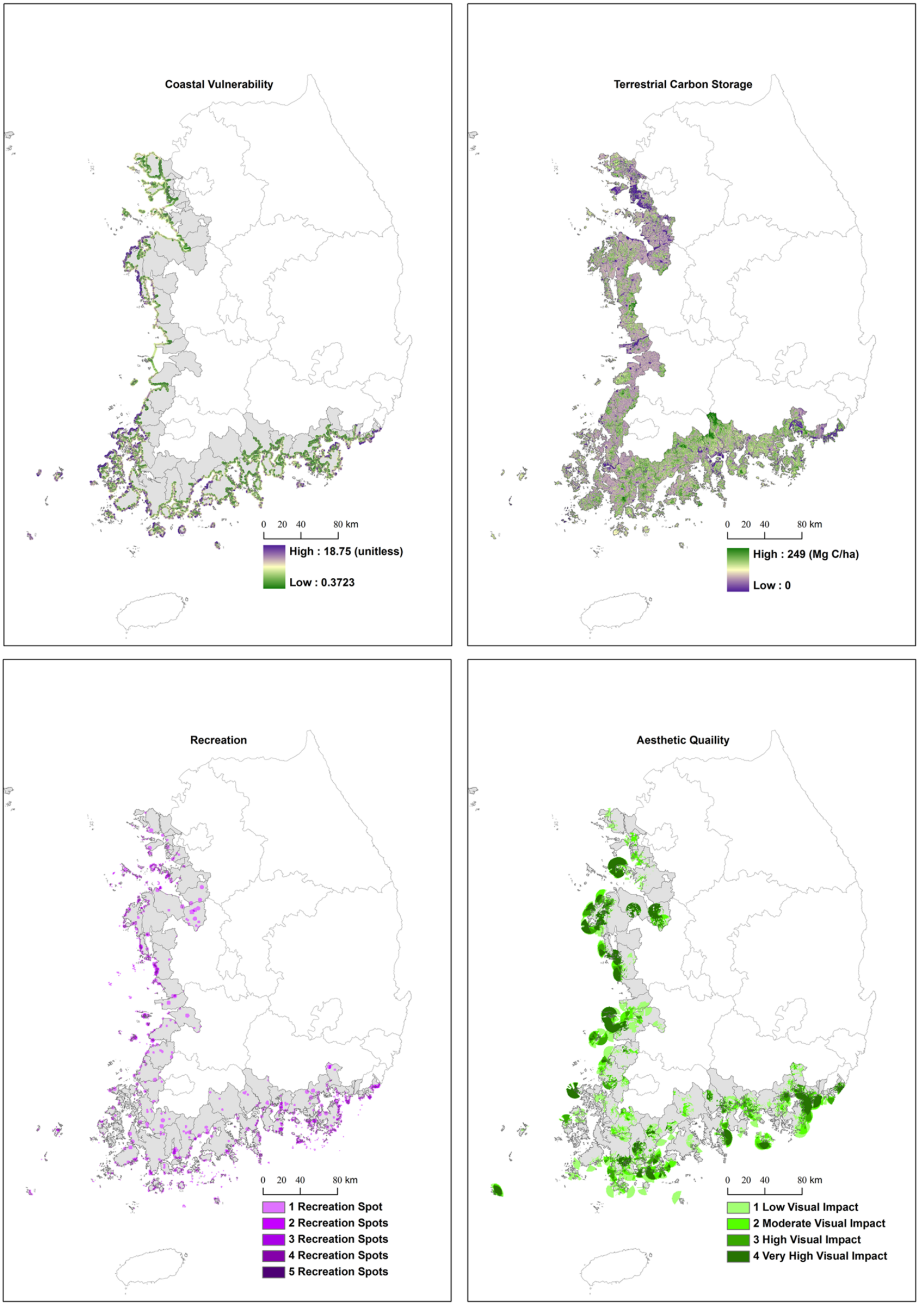
Ecosystem services assessments of coastal areas in South Korea. Feature values of coastal vulnerability and terrestrial carbon storage are expressed in continuous values from poor (purple) to good (green) conditions. In addition, recreation and aesthetic quality are classified as low, moderate, high, or very high.

Fig 3 exhibits four ecosystem services: coastal vulnerability, carbon storage, recreation, and aesthetic quality. The spatial distribution of each ecosystem service is distinctly different throughout the coastal counties. By mapping coastal vulnerability, we identify the locations of low or high vulnerability. The west coast of Korea is more vulnerable to climate change and inundations than the south coast. The map of terrestrial carbon storage displays the location of high quantities of carbon storage. According to the map, the western counties are associated with low levels of carbon storage, whereas the most southern counties have the high levels of carbon storage. Northwestern counties are located near Seoul (the capital of South Korea), and they have rapidly developed for urbanization and industrialization under national policies. Southwestern counties have reclaimed coastal wetlands as large-scale reclamation projects (e.g., Saemangeum project) to stimulate the regional economy in a short period. Thus, western counties have deteriorated natural habitats and led to the low levels of carbon storages. However, national economic policies have not considered southern counties for the development, as the counties are traditionally agricultural and fishing areas.

Maps of recreation and aesthetic represent the number of recreational sites and visual impacts in the study area. The number of recreational sites indicates the intensity of human recreational uses in the coastal areas, and the aesthetic quality map demonstrates the specific areas of aesthetic appreciation. The locations of high recreational sites and aesthetic quality are evenly distributed across the counties. Many counties are generally of high value to multiple services with good habitat conditions (Fig 3). However, Busan, the second largest metropolis in South Korea, has undergone a high level of habitat risk due to anthropogenic impacts, but the county still provides much recreation and aesthetic quality (Figs 2 and 3).

We performed correlation analysis to verify the accuracy of the spatial assessments (see methods). Coastal habitat risk assessment was negatively associated with species diversity (ρ = -0.158), habitat diversity (ρ = -0.346), and ecological health (ρ = -0.112). The coastal vulnerability was positively associated with the grades of typhoon damages (ρ = 0.297). While recreation assessment was negatively correlated with the grades for coastal recreation and tourism (ρ = -0.240), the aesthetic quality was positively correlated with the grades (ρ = 0.296). When counties had more recreation spots from the recreation assessment, they also tended to have a higher status (lower grade) for coastal recreation and tourism, estimated by the Korean government. However, counties with a higher aesthetic quality tended to have a lower status (higher grade) for recreation and tourism. The grades of coastal recreation and tourism published by the Korean government considered a direct human use only such as sea bathing or recreational fishing. Thus, the grade systems may not reflect the values of viewpoints located in pristine areas where aesthetic values are high but direct human usage is minimal. Therefore, counties with high aesthetic quality tended to have a low status of coastal recreation and tourism. In addition, the areas (km2) of the first grade of ecological zoning map and protected areas were positively associated with total carbon storage (R = 0.594) and negatively associated with terrestrial habitat risk (R = -0.580). All correlation results had a significant P-value (P<0.05).

### Synergies and tradeoffs of habitat risk and ecosystem services

Most of the habitat risks and ecosystem services significantly correlate with one another (Table 2). We found significant positive relations between cultural services and between habitat risks: recreation and aesthetic quality are strongly correlated (r = 0.7–0.9), and coastal habitat risk and terrestrial habitat risk are also moderately correlated (r = 0.4–0.6). In particular, the coastal habitat risk is negatively correlated with regulation and cultural services and the terrestrial habitat risk is also negatively correlated with them except for coastal vulnerability (P<0.01). Aquaculture productions are insignificant with both coastal and terrestrial habitat risk, while aquaculture productions are positively correlated with regulation and cultural services. Furthermore, carbon storage is positively correlated with cultural services, i.e., recreation and aesthetic quality.

**Table 2:** Pearson’s correlation analysis between habitat risk assessments and ecosystem services.

Variable	Habitat Risk		Provisioning	Regulating		Cultural	
	Coastal habitat risk	Terrestrial habitat risk	Aquaculture	Coastal vulnerability	Carbon storage	Recreation	Aesthetic quality
**Coastal habitat risk**	1						
**Terrestrial habitat risk**	**0.502** **	1					
**Aquaculture**	-0.149	-0.240	1				
**Coastal vulnerability**	**-0.437** **	-0.141	0.099	1			
**Carbon storage**	**-0.346** **	**-0.515** **	**0.278** *	0.177	1		
**Recreation**	**-0.351** **	**-0.453** **	**0.648** **	0.176	**0.448** **	1	
**Aesthetic quality**	-0.257	**-0.394** **	**0.539** **	0.189	**0.338** *	**0.801** **	1

* P<0.05,

** P<0.01

The results of the t-test are presented in Table 3. The statistical results show that there are significant differences in the values of the habitat risks and four ecosystem services (aquaculture, carbon storage, recreation, and aesthetic quality) between reclamation-dominated and conservation-dominated counties. The intensities of terrestrial and coastal habitats are significantly greater in the reclamation-dominated counties (P <0.01). In contrast, aquaculture productions and the numbers of recreation activities and aesthetic quality are significantly larger in the conservation-dominated counties (P<0.01). While the carbon storage of regulation services is significantly higher in the conserved counties, coastal vulnerability is not different between the two groups.

**Table 3:** Ecosystem Services Characteristics of reclamation-dominated and conservation-dominated counties.

Independent variable	Reclamation^†^	Conservation^‡^	F-test^¶^	Adjusted T-test^§^
	(n = 34)	(n = 25)		
Coastal habitat risk	5.57 (2.39)	3.09 (1.71)	19.56**	-4.65**
Terrestrial habitat risk	120.6 (74.43)	46.02 (38.58)	20.93**	-5.00**
Aquaculture	59.32 (180.27)	1683.48 (3493.99)	5.64*	2.73*
Coastal vulnerability	4.13 (2.14)	4.43 (1.15)	0.4	0.67
Carbon storage	65.39 (27.23)	90.49 (16.01)	16.89**	4.43**
Recreation	200.81 (177.72)	513.9 (374.1)	14.32**	4.43**
Aesthetic value	1,278.55 (1,432.79)	2,609.48 (2,761.83)	4.69**	2.30**

^**†**^ Means and Standard Deviance (in parentheses) of reclamation-dominated counties.

^‡^ Means and Standard Deviance (in parentheses) of conservation-dominated counties.

^**¶**^ F-statistics is testing variance equality of reclamation-dominated or conservation-dominated counties.

^**§**^ Adjusted t-test is used when the variances are not equal.

* P<0.05,

** P<0.01

### Social and ecological impacts on the designation of conservation and reclamation

A comparison of the demographic, socioeconomic, and biophysical predictors between conservation-dominated counties and reclamation-dominated counties is shown in Table 4. The reclamation-dominated counties are significantly larger than conservation-dominated counties in population, population density, household, family size, appraised value of land, and GRDP (P<0.01). On the contrary, the reclaimed counties are significantly smaller in the average age of population, fishery households, and median slope of coastline (P <0.05) (S6 Table).

**Table 4:** Negative binomial model results for conservation-dominated and reclamation-dominated counties.

Independent variable	Protected Area (ha)		Reclaimed Area (ha)		
	Model1	Model4	Model2	Model5	Model8
Constant	6.13* (3.38)	8.03*** (0.62)	11.23*** (2.40)	8.60*** (0.54)	8.45*** (0.57)
Population, 1000 persons	0.067** (0.033)	0.076*** (0.026)	-0.053** (0.023)		-0.042* (0.024)
Population density, 100 persons/m^2^	-0.009 (0.013)		-0.017* (0.009)	-0.018*** (0.004)	
Household, 100 families	-0.018* (0.009)	-0.021*** (0.007)	0.014** (0.007)		0.012* (0.007)
Average age	0.033 (0.063)		-0.058 (0.046)		
Land value, thousand won/m^2^	-0.006*** (0.002)	-0.008*** (0.001)	-7.3e-4 (0.002)		-2.9e-3*** (0.001)
GRDP, billion won	-1.4e-4* (0.000)	-1.3e-4* (0.000)	1.3e-4* (0.000)	7.4e-5** (0.000)	1.9e-4*** (0.000)
Fishery households, 10 families	0.008*** (0.003)	0.008*** (0.003)	0.003 (0.002)	0.002 (0.002)	0.003 (0.002)
Tourist, 1000 persons	-3e-5 (0.000)	-4e-5 (0.000)	1.3e-6 (0.000)	3.5e-5 (0.000)	1.8e-5 (0.000)
Slope, degree	0.375*** (0.141)	0.356** (0.141)	-0.395*** (0.102)	-0.331*** (0.103)	-0.385*** (0.100)
Dispersion	1.640	1.652	1.127	1.223	1.233
AIC	925.30	921.80	974.01	971.63	976.20
Pearson Chi-square	52.048	51.416	57.457	56.445	48.458
DF	45	47	45	49	47
Deviance/DF	1.157	1.094	1.277	1.152	1.031
p-value	0.219	0.305	0.101	0.217	0.414
Condition Index	142.724	8.216	142.724	6.656	8.216

* P<0.10,

** P<0.05,

*** P<0.01.

Standard errors are in parentheses.

Two generalized linear models revealed a significant association between socioeconomic/biophysical factors and reclaimed or protected areas at a county level (Table 4). The model for protected areas has a positive association with protected areas with population, fishery households, and biophysical slope (P<0.05), but have a negative association with the number of households and land values (P<0.01). The most significant factors associated with conservation are demographic factors and land values. The model for reclaimed areas has a positive association with reclaimed areas with GRDP (P<0.01), while the model has a negative association with land values and biophysical slope (P<0.01). The most significant factors with reclamation are economic and biophysical characteristics.

## Discussion

In this study, the ecosystem services approach that combines spatial assessment with statistical analysis provided spatially explicit information on habitat risks and ecosystem services along with synergies and tradeoffs among habitat risks and ecosystem services. Additionally, we determined drivers on the designation of reclamation projects or conservation policies by using a generalized linear model. We showed how demographic, socioeconomic, and biophysical factors associated with the designation of reclamation and conservation areas at the county level. Thus, the approach can provide scientific evidence that previous studies have not treated for a decision-making process in coastal managements. Also, the benefit of the approach is to spend no additional cost, because the necessary data has been established and verified by Korean governments. Indeed, the approach is available to adopt immediately in coastal areas where it need to analysis for development or conservation.

Our results indicated that reclamation of coastal wetlands has severely reduced biodiversity and their ecosystem services. The statuses of biodiversity and associated ecosystem services in conservation-dominated counties were quantitatively better than reclamation-dominated counties. Previous studies suggested that coastal wetland reclamation leads to degrade ecological health and biodiversity in coastal areas [7, 8]. Furthermore, we showed that reclamation deteriorated regulating and cultural ecosystem services and decreased the benefits of the services to human societies.

Whereas economic factors were highly associated with reclamation areas at a county level, habitat conditions and demographic factors are significantly associated with protected areas. Conventionally, when coastal counties have been urbanized, development pressures on coastal wetlands have been increased for further regional economic growth [9]. Along with the information, our results suggested that rapidly urbanized counties but not yet extremely high population densities suffered higher pressure for reclamation area. In addition, as the designation criteria of protected areas in South Korea are high biodiversity, threatened native species, and representative natural landscape [19], the result which good coastal habitat conditions associated with protected areas is reasonable. Rural coastal counties have experienced rapid aging societies and decreased population because of migration from rural to urban areas since the early 1960s [49]. It means that the number of stakeholders in rural coastal counties is relatively small and thus Korean government and stakeholders have been easily reaching consensus for compensation and resolved several conflicts in the designation of protected areas.

### The Status and Potential Risks of Coastal Areas

Spatially mapped ecosystem services can be used to select possible development sites (e.g., for industrialization or urbanization) that minimize degradation of ecosystem services [61, 62]. In addition, the spatial assessments allow decision makers to prioritize the areas that should be included in conservation programs to protect from potential development [43, 63]. In particular, ocean national parks in South Korea have preserved their habitats in excellent condition and seem to have high biodiversity than those areas outside of protected areas (Figs 1 and 2). Even though Busan has undergone severe habitat risk and biodiversity loss in both terrestrial and coastal areas, the areas still provide extremely high aesthetic quality and multiple recreation activities. The levels of coastal and terrestrial habitat risks have a negative impact on coastal vulnerability, carbon storage, recreation, and aesthetic quality, indicating that development projects degrade and change the status of cultural services. Therefore, many recreational activities and high aesthetic quality in Busan may deteriorate in the near future.

Provisioning services are affected by habitat conditions and regulation services [64], and also influence cultural services by forming tradeoffs among them [65]. In this study, aquaculture activities are negatively correlated to cultural ecosystem services. Since aquaculture activities cause the degradation of both coastal and terrestrial habitats by using intensified feeds and facilities, the correlation between aquaculture and habitat risks is not significant (Table 2). The coastal fishery of South Korea largely divides into two categories: adjacent waters fisheries and shallow sea cultures. While the production of shallow sea cultures have dramatically raised from 772,731 metric tons in 1990 to 1,515,210 metric tons in 2013, the fishery production of adjacent waters fisheries have decreased from 1,471,810 metric tons in 1990 and 1,044,697 metric tons in 2013 [49]. Most aquaculture facilities are located on the southwest coast of South Korea due to good habitat conditions. The increases of the facilities can negatively impact the aesthetic amenities [28], as the aesthetic quality of this area is very high. If sufficient information had been available regarding tradeoffs among ecosystem services or between biodiversity and ecosystem services, the impact of several threats could have been reduced by compensating a loss in one service (e.g., food provision) with gains in another service (e.g., aesthetic values) through conservation strategies [48].

### Coastal Conservation Strategies

Consideration of spatial scale differences between an ecological scale and an institutional scale is crucial for implementing conservation strategies [56]. An ecological scale indicates that biodiversity and ecosystem services are supplied, and an institutional scale is a basic unit for implementing a management plan [3]. In South Korea, reclamation projects and conservation policies have implemented and performed at the county level, as local governments have priority to establish and implement coastal management plans according to the Coast Management Act. These policies have caused change not only to natural systems but also to human society. For example, protected areas prevent the extraction of provisioning ecosystem services and the restrict development [30]. On the other hand, local people of reclaimed areas are forced to immigrate to other sites, because the reclamation has permanently destroyed natural habitats necessary for fisheries [66]. The comprehensive involvement of social and ecological characteristics within conservation strategies could minimize the conflicts between conservation and development aims [67]. Within this context, our results show that the status of habitat risks and ecosystem services are distinctively different between conservation-dominated and reclamation-dominated counties (Table 3). Protected counties sustain better habitat conditions and higher levels of ecosystem services than reclaimed counties. Furthermore, the status of coastal vulnerability is not significantly different between conservation and reclamation-dominated counties (Table 3). This suggests that artificially constructed structure and natural habitats in estuaries may play a similar role in protecting coastal zones. Most urban areas are well protected from coastal disasters such as storms and floods by constructing horizontal levees, while these areas have few recreational spots compared to protected areas.

The reclamation and conservation policies also influence social and biophysical characteristics at a county level. Our ANOVA results show that local society and natural environments may be of relevance for the designations of the conservation and development policies (Tables 3 and 4). Additionally, demographic pressures and habitat conditions of coastal areas would affect the designation of conservation (Table 4). The number of fishery households and the coastal slope may have positive associations with the designation of conservation areas. The number of fishery households may be closely linked with the habitat conditions of coastal wetlands, because the better habitat conditions allow the households to obtain higher fishery productivity. In addition, the steeper slope constrains the development of areas with high costs and can avoid reclamation or other development projects. However, the reclamation projects would strongly depend on economic efficiency. The land values and coastal slope negatively relate to the areas of reclamation. The cheap land value allows investors to perform coastal development with low costs, and the flatter slope guarantees reclamation without high civil engineering techniques. Although most social and biophysical determinants are significantly different between reclaimed and protected counties, the number of tourists is not different between the two groups. This indicates that tourists may be affected more by the distance from their residences or good traffic infrastructures than the level of recreational spots and aesthetic quality during the selection process. To establish comprehensive conservation strategies in coastal areas, decision-makers need to overcome this trend.

If future research confirms a causal relationship between socioeconomic characteristics and the designation of reclamation projects or conservation policies, the coefficient estimates in Table 4 may be used to predict the designation of protected areas or reclaimed areas for different biophysical and socioeconomic predictors. For example, larger population and fishery households lead to greater protected areas and lower reclamation at the county level; while greater households and GRDP led to lower protection and greater reclamation and the appraised value of land is negatively related to both the designation of conservation and selection of reclamation areas. Also, areas with steeper slopes in coastal counties are more likely to be designated as a conservation area and areas with flatter slopes are more likely to be selected as reclamation projects (Table 4). As predicted by the model, if the population increases by ten thousand, households increase by 3000, and other predictors are constant in Geoje-si, then the area of conservation is predicted to 27,270 ha (originally 18,583 ha) and the area of reclamation is predicted to be 849 ha (originally 1,571 ha). Therefore, we can expect that there may no longer be reclamation projects in Geoje-si.

As seen in Fig 1, the locations of protected areas in coastal zones depart from urbanized areas. The protected areas in South Korea provide high biodiversity and multiple ecosystem services as a result of the conservation purposes (Figs 2 and 3, Table 3), but outside of protected areas there is no legal basis to restrain effectively development and reduce the degradation of environmental conditions from human activities. Since cultural services (recreational activities and aesthetic quality) distribute evenly throughout the coastal areas of South Korea, even in highly urbanized areas, employing cultural services can support coastal conservation and sustainable development outside of protected areas [46]. Indeed, cultural ecosystem services are used explicitly by humans and generate benefits for local communities [68]. The benefits have strongly motivated local people to maintain natural habitats and related ecosystem services by owning, managing, and conserving areas [69], as the cultural services are provided by biodiversity and are sometimes linked with provisioning ecosystem services at a regional level [3].

### Future Works

Natural habitats and ecosystem services are to change and transform over time with human activities. As such, the construction of scenarios that reflect coastal planning zone systems is necessary to implement effectively conservation policy and predict the impacts of policy by using the ecosystem services approach [70]. This study did not explore time-series but rather focused on the period around 2010, so the results cannot anticipate spatial changes in biodiversity and ecosystem services over time. Further studies should employ more advanced spatial models to estimate the spatial variation of biodiversity and ecosystem services by altering inputs in accordance with management scenarios. Investigating tradeoffs among scenarios or between temporal scales would enable decision makers to maximize the quality and quantity of ecosystem services [71, 72].

Field works are crucial in elaborating spatial assessments and statistical models at the local and regional levels [37, 73]. Using the results of the field works, we can improve the accuracy and reliability of spatial assessments and establish accurate statistical models. First, ecological field works allow us to perform the accuracy assessments for the spatial models of habitat risks and ecosystem services. In the field, researchers can adopt similar methods of the assessments for land cover classification in remote sensing [73]. For example, using the map of recreation assessments (Fig 3), field workers will verify the recreation spots in the coastal areas with GPS devices. In addition, sampling and experiments in the fields can be an example for the accuracy assessments for the spatial models. For example, researchers collected soil samples in coastal wetlands and performed field and laboratory experiments to figure out the sediment biogeochemistry of coastal habitats [57]. Using the biogeochemical results, researchers graded the status of biodiversity and ecological health at each sample points. Then, we compared the coastal grades from field works with the result of coastal habitat risk assessment.

Second, sociological field works based on surveys and interviews help to construct an accurate statistical model [37]. To increase the efficiency of the field works, researchers can use the results of existing models. For example, we can calculate the errors in the models as a difference between the actual values of protected or reclaimed areas and the predicted values from the generalized linear models in each county (Table 4). By identifying positive and negative outliers, we can select specific counties where need to collect additional information from the field works. The information helps to figure out necessary variables to improve the accuracy of the statistical models. As followed this process, researchers will focus on the outlier counties for the field works, not all counties. Thus, they can save time and resources during the studies.

The ecosystem services approach in this study provided opportunities to constitute guidelines for conservation and sustainable development of biodiversity and ecosystem services. We assessed the relative risk of human activities and regulation and cultural services in coastal areas. The results of the statistical analyses provide synergies and tradeoffs among services and between habitat risks and services. Additionally, social and ecological characteristics in the unified spatial scale correlated with the designation of reclamation and conservation areas. We expect that the findings can help decision makers determine priority areas for conservation outside of protected areas.

China and other south-Asia countries have followed similar economic development model to South Korea; import substitution industrialization in heavy industry, governmental control over finance and support for enterprises, dependence on export markets [74]. Therefore, rapid developments in coastal areas of South Korea is spreading to East-Asian and southern Asian countries where have experienced rapid economic growths for last decade [5]. For example, China has reclaimed coastal wetlands 60,000 ha per year from 2010 to 2020 for infrastructure development [5]. Thus, this study will provide a valuable perspective to achieve both sustainable development and conservation in Asia countries.

## Supporting Information

S1 Table The types and protective distance of coastal habitats and recreational activities (DOCX)

S2 Table The threats and ‘the maximum distance of each threat effect’* on habitat quality (DOCX)

S3 Table Estimates of carbon storage (MgC/ha) in four carbon pools (aboveground biomass, belowground biomass, soil organic matter and dead organic matter) associated with land use and land change type (DOCX)

S4 Table Land use and land cover type and sensitivity of LULC types to each threat (DOCX)

S5 Table Variables in the negative binomial model for areas of conservation and reclamation at county level (DOCX)

S6 Table Socioeconomic and biophysical characteristics of reclamation-dominated and conservation-dominated counties (DOCX)
