# Supplementary material for: Assessment of Coastal Ecosystem Services for Conservation Strategies in South Korea
Source: PLoS One. 2015 Jul 29;10(7):e0133856. doi: 10.1371/journal.pone.0133856 (PMC4519238; doi:10.1371/journal.pone.0133856)
Supplement: S1 Table — (DOCX) [file pone.0133856.s001.docx]

**S1 Table. Type and protective distance of coastal habitats and type and buffer distance of recreational activities**

| **Name** | **Explanation** | **Distance (m)** | **Note** |
| --- | --- | --- | --- |
| Tideflat | Tidal flat | 1500 | Protection |
| Sand | Sand flat | 500 |  |
| Msg | Mix flat | 500 |  |
| Gravel | Gravel | 300 |  |
| Rocky | Rocky | 1000 |  |
| Beaches | Sea bathing | 500 | Recreation |
| Fishing | Sea fishing | 300 |  |
| Seavillage | Fishery experience village | 1500 |  |
| Visitingcenter | Coastal visitors’ center | 1000 |  |
| Tourism | Tourist attraction | 2000 |  |
| Yort | Yacht | 2000 |  |
| Sightingc | Scenic view | 1000 |  |
