# Supplementary material for: Assessment of Coastal Ecosystem Services for Conservation Strategies in South Korea
Source: PLoS One. 2015 Jul 29;10(7):e0133856. doi: 10.1371/journal.pone.0133856 (PMC4519238; doi:10.1371/journal.pone.0133856)
Supplement: S2 Table — (DOCX) [file pone.0133856.s002.docx]

**S2 Table. The threats and ‘the maximum distance of each threat effect’* on habitat quality**

| **Threat** | **Explanation** | **Max_Dist (m)** | **Note** |
| --- | --- | --- | --- |
| Prds | Primary road | 3000 | Terrestrial |
| Srds | Second road | 1000 |  |
| Rails | Rail | 1000 |  |
| Urb | Urban area | 10000 |  |
| Crp | Paddy, field and orchard | 5000 |  |
| Grnh | Other farmland | 3000 |  |
| Indc | Industrial area | 10000 |  |
| Nprt | Port and harbor | 5000 |  |
| Recl | Reclamation area | 8000 |  |
| Aquaculture | Aquaculture facility | 300 | Coastal |
| Industry | Industrial complex | 1500 |  |
| Port | Trade port | 1000 |  |
| Nuclear | Nuclear power plant | 2000 |  |
| Reclamation | Reclamation area | 1000 |  |
| National | National port | 500 |  |
| Harbor | National harbor |  |  |
| RecFishing | Sea fishing | 200 |  |
| RecBeach | Sea bathing | 300 |  |

*‘Maximum distance of each threat effect’ means that the distance from each threat within which habitat quality is affected.
