# Supplementary material for: Assessment of Coastal Ecosystem Services for Conservation Strategies in South Korea
Source: PLoS One. 2015 Jul 29;10(7):e0133856. doi: 10.1371/journal.pone.0133856 (PMC4519238; doi:10.1371/journal.pone.0133856)
Supplement: S3 Table — (DOCX) [file pone.0133856.s003.docx]

**S3 Table. Estimates of carbon storage (MgC/ha) in four carbon pools (aboveground biomass, belowground biomass, soil organic matter and dead organic matter) associated with land use and land change type**

| **LULC** | **Explanation** | **C_above** | **C_below** | **C_soil** | **C_dead** |
| --- | --- | --- | --- | --- | --- |
| 110 | Residential | 0.00 | 0.00 | 0.00 | 0.00 |
| 120 | Industrial | 0.00 | 0.00 | 0.00 | 0.00 |
| 130 | Commercial | 0.00 | 0.00 | 0.00 | 0.00 |
| 140 | Recreational | 0.00 | 0.00 | 0.00 | 0.00 |
| 150 | Traffic | 0.00 | 0.00 | 0.00 | 0.00 |
| 160 | Public facilities | 0.00 | 0.00 | 0.00 | 0.00 |
| 210 | Paddy | 0.00 | 0.00 | 67.70 | 0.00 |
| 220 | Field | 0.00 | 0.00 | 62.20 | 0.00 |
| 230 | Green house | 0.00 | 0.00 | 45.90 | 0.00 |
| 240 | Orchard | 0.00 | 0.00 | 51.00 | 13.00 |
| 250 | Other farmland | 0.00 | 0.00 | 45.90 | 0.00 |
| 310 | Broadleaf forest | 19.93 | 8.17 | 51.00 | 13.00 |
| 311 | 1^st^ age-class | 19.93 | 8.17 | 51.00 | 13.00 |
| 312 | 2^nd^ age-class | 40.66 | 16.67 | 66.00 | 13.00 |
| 313 | 3^rd^ age-class | 69.37 | 28.44 | 63.00 | 13.00 |
| 314 | 4^th^ age-class | 89.34 | 36.63 | 96.00 | 13.00 |
| 315 | 5^th^ age-class | 110.85 | 45.45 | 81.00 | 13.00 |
| 316 | 6^th^ age-class | 110.85 | 45.45 | 67.00 | 13.00 |
| 320 | Coniferous forest | 32.51 | 9.10 | 51.00 | 22.00 |
| 321 | 1^st^ age-class | 32.51 | 9.10 | 51.00 | 22.00 |
| 322 | 2^nd^ age-class | 42.60 | 11.93 | 66.00 | 22.00 |
| 323 | 3^rd^ age-class | 51.57 | 14.44 | 63.00 | 22.00 |
| 324 | 4^th^ age-class | 61.52 | 17.23 | 96.00 | 22.00 |
| 325 | 5^th^ age-class | 71.75 | 20.09 | 81.00 | 22.00 |
| 326 | 6^th^ age-class | 71.75 | 20.09 | 67.00 | 22.00 |
| 330 | Mixed forest | 21.03 | 7.25 | 51.00 | 17.50 |
| 331 | 1^st^ age-class | 21.03 | 7.25 | 51.00 | 17.50 |
| 332 | 2^nd^ age-class | 37.63 | 12.98 | 66.00 | 17.50 |
| 333 | 3^rd^ age-class | 58.58 | 20.21 | 63.00 | 17.50 |
| 334 | 4^th^ age-class | 73.14 | 25.23 | 96.00 | 17.50 |
| 335 | 5^th^ age-class | 91.82 | 31.68 | 81.00 | 17.50 |
| 336 | 6^th^ age-class | 91.82 | 31.68 | 67.00 | 17.50 |
| 410 | Natural grassland | 4.17 | 16.69 | 88.20 | 0.00 |
| 420 | Golf course | 1.15 | 4.58 | 11.50 | 0.00 |
| 430 | Other grassland | 1.88 | 7.53 | 42.40 | 0.00 |
| 510 | Inland wetland | 0.00 | 0.00 | 88.00 | 11.00 |
| 520 | Coastal wetland | 0.00 | 0.00 | 0.00 | 0.00 |
| 610 | Mining area | 0.00 | 0.00 | 0.00 | 0.00 |
| 620 | Other barren | 1.28 | 5.13 | 42.40 | 0.00 |
| 710 | Inland water | 0.00 | 0.00 | 0.00 | 0.00 |
| 720 | Salt water | 0.00 | 0.00 | 0.00 | 0.00 |
| 990 | No value | 0.00 | 0.00 | 0.00 | 0.00 |

Carbon storage data are collected from local studies and conducted for the IPCC [53-55]. Here, carbon storage is measured from the sea surface to a depth of 1 m.
