# Supplementary material for: Assessment of Coastal Ecosystem Services for Conservation Strategies in South Korea
Source: PLoS One. 2015 Jul 29;10(7):e0133856. doi: 10.1371/journal.pone.0133856 (PMC4519238; doi:10.1371/journal.pone.0133856)
Supplement: S4 Table — (DOCX) [file pone.0133856.s004.docx]

**S4 Table. Land use and land cover type and sensitivity of LULC types to each threat**

| **LULC** | **Name** | **Habitat** | **L_prds** | **L_srds** | **L_rails** | **L_urb** | **L_crp** | **L_grnh** | **L_indc** | **L_nprt** | **L_recl** |
| --- | --- | --- | --- | --- | --- | --- | --- | --- | --- | --- | --- |
| 110 | Residential | 0 | 0 | 0 | 0 | 0 | 0 | 0 | 0 | 0 | 0 |
| 120 | Industrial | 0 | 0 | 0 | 0 | 0 | 0 | 0 | 0 | 0 | 0 |
| 130 | Commercial | 0 | 0 | 0 | 0 | 0 | 0 | 0 | 0 | 0 | 0 |
| 140 | Recreational | 0.1 | 0.3 | 0.2 | 0.2 | 0.3 | 0 | 0 | 0.3 | 0 | 0 |
| 150 | Traffic | 0.1 | 0.3 | 0.2 | 0.2 | 0.3 | 0 | 0 | 0.3 | 0 | 0 |
| 160 | Public facilities | 0.2 | 0.4 | 0.3 | 0.3 | 0.4 | 0 | 0 | 0.4 | 0 | 0 |
| 210 | Paddy | 0.5 | 0.3 | 0.2 | 0.2 | 0.9 | 0 | 0.1 | 1 | 0.9 | 0 |
| 220 | Field | 0.7 | 0.5 | 0.4 | 0.4 | 0.9 | 0 | 0.1 | 1 | 0.9 | 0 |
| 230 | Green house | 0.2 | 0.3 | 0.2 | 0.2 | 0.8 | 0.1 | 0 | 0.9 | 0.8 | 0 |
| 240 | Orchard | 0.4 | 0.3 | 0.2 | 0.2 | 0.9 | 0 | 0.1 | 1 | 0.9 | 0 |
| 250 | Other farmland | 0.4 | 0.3 | 0.2 | 0.2 | 0.8 | 0.1 | 0 | 0.9 | 0.8 | 0 |
| 310 | Broadleaf forest | 1 | 1 | 0.9 | 0.9 | 0.9 | 1 | 1 | 1 | 0.9 | 1 |
| 320 | Coniferous forest | 1 | 1 | 0.9 | 0.9 | 0.9 | 1 | 1 | 1 | 0.9 | 1 |
| 330 | Mixed stand forest | 1 | 1 | 0.9 | 0.9 | 0.9 | 1 | 1 | 1 | 0.9 | 1 |
| 410 | Natural grassland | 1 | 0.5 | 0.4 | 0.4 | 0.6 | 0.4 | 0.5 | 0.7 | 0.6 | 1 |
| 420 | Golf course | 0.5 | 0.2 | 0.1 | 0.1 | 0.2 | 0.1 | 0.1 | 0.3 | 0 | 0 |
| 430 | Other grassland | 0.7 | 0.3 | 0.2 | 0.2 | 0.4 | 0.3 | 0.3 | 0.5 | 0.4 | 1 |
| 510 | Inland wetland | 1 | 0.7 | 0.6 | 0.6 | 1 | 0.8 | 0.9 | 1 | 1 | 1 |
| 520 | Coastal wetland | 1 | 0.7 | 0.6 | 0.6 | 1 | 0.8 | 0.9 | 1 | 1 | 1 |
| 610 | Mining area | 0.3 | 0.2 | 0.1 | 0.1 | 0.1 | 0.1 | 0.1 | 0.1 | 0 | 0 |
| 620 | Other barren | 0.6 | 0.3 | 0.2 | 0.2 | 0.4 | 0.3 | 0.3 | 0.4 | 0 | 1 |
| 710 | Inland water | 0 | 0 | 0 | 0 | 0 | 0 | 0 | 0 | 0 | 0 |
| 720 | Salt water | 0 | 0 | 0 | 0 | 0 | 0 | 0 | 0 | 0 | 0 |
|  | No value |  |  |  |  |  |  |  |  |  |  |

Each LULC habitat score, taken from data extracted from an Ecological Zoning Map [21], is assigned a value from 0 to 1. The relative sensitivity of each habitat type to each threat is estimated using the Environmental Conservation Value Assessment Map [21].
