# Supplementary material for: Assessment of Coastal Ecosystem Services for Conservation Strategies in South Korea
Source: PLoS One. 2015 Jul 29;10(7):e0133856. doi: 10.1371/journal.pone.0133856 (PMC4519238; doi:10.1371/journal.pone.0133856)
Supplement: S5 Table — (DOCX) [file pone.0133856.s005.docx]

**S5 Table. Variables in the Negative binomial model for areas of conservation and reclamation at county level**

| **Variable** | **Description** | **Mean** | **SD** |
| --- | --- | --- | --- |
| Protected area, Y_1_ | protection areas at coastal counties (ha) | 7,197.8 | 15,088.7 |
| Reclamed area, Y_2_ | reclamation areas at coastal counties (ha) | 3,443.8 | 5,653.4 |
| Population, X_1_ | population of coastal counties | 167,776.3 | 138,669.7 |
| Population density, X_2_ | population density | 2,670 | 4,389 |
| Household, X_3_ | the number of households | 62,268 | 47,988 |
| Family size, X_4_ | average number of household members | 2.84 | 0.73 |
| Average age, X_5_ | average age of population | 42.6 | 6.3 |
| Land value, X_6_ | appraised value of land | 257,432 | 249,049 |
| GRDP, X_7_ | gross regional domestic product | 4,442,341 | 4,611,934 |
| Fishery households, X_8_ | the number of fishery households | 907 | 1094 |
| Tourist, X_9_ | the number of tourists | 4,823,809 | 4,738,776 |
| Slope, X_10_ | the average coastal slope within 1km | 3.45 | 1.87 |
