# Supplementary material for: Assessment of Coastal Ecosystem Services for Conservation Strategies in South Korea
Source: PLoS One. 2015 Jul 29;10(7):e0133856. doi: 10.1371/journal.pone.0133856 (PMC4519238; doi:10.1371/journal.pone.0133856)
Supplement: S6 Table — (DOCX) [file pone.0133856.s006.docx]

**S6 Table. Socioeconomic and biophysical characteristics of reclamation dominated and conservation dominated counties**

| **Independent variable** | **Reclamation^†^**  **(n=34)** | | **Conservation**^‡^  **(n=25)** | | **F-test^¶^** | **Adjusted**  **T-test^§^** |
| --- | --- | --- | --- | --- | --- | --- |
| Population, persons | 220,995 | (145,584) | 95,398 | (88,649) | 14.59** | -4.1** |
| Population density | 4,254.3 | (5,087.8) | 516 | (1,602.2) | 12.53** | -4.02** |
| Household | 80,843 | (50,131.3) | 37,006 | (30,921.7) | 14.9** | -4.14** |
| Family size, persons | 3.06 | (0.83) | 2.54 | (0.44) | 8.23** | -3.13** |
| Average age | 39.67 | (4.62) | 46.50 | (6.16) | 23.74** | 4.66** |
| Land value, won/m^2^ | 370,620 | (261,273) | 103,497 | (114,891) | 22.8** | -5.3** |
| GRDP, million won | 5,897,360 | (5,018,894) | 2,463,517 | (3,112,708) | 9.1** | -3.23** |
| Fishery households | 503.1 | (627.2) | 1,456.5 | (1,344.3) | 13.25** | 3.29** |
| Tourist, persons | 5,148,040 | (5,758,002) | 4,405,012 | (3,025,386) | 0.33 | 0.62 |
| Slope, degree | 3.041 | (1.915) | 4.011 | (1.684) | 4.08* | 2.06* |

**^†^** Means and Standard Deviance (in parentheses) of reclamation dominated counties.

^‡^ Means and Standard Deviance (in parentheses) of conservation dominated counties.

**^¶^** F statistics testing variance equality of reclamation dominated or conservation dominated counties.

**^§^** Adjusted t-test is used when the variances are not equal.

* P<0.05, ** P<0.01
